# Influence of Oral Anticoagulation and Antiplatelet Drugs on Outcome of Elderly Severely Injured Patients

**DOI:** 10.3390/jcm10081649

**Published:** 2021-04-13

**Authors:** Maximilian Kerschbaum, Siegmund Lang, Leopold Henssler, Antonio Ernstberger, Volker Alt, Christian Pfeifer, Michael Worlicek, Daniel Popp

**Affiliations:** 1 Department of Trauma Surgery, University Medical Centre Regensburg, D-93053 Regensburg, Germany; maximilian.kerschbaum@ukr.de (M.K.); siegmund.lang@ukr.de (S.L.); leopold.henssler@ukr.de (L.H.); volker.alt@ukr.de (V.A.); christian.pfeifer@ukr.de (C.P.); michael.worlicek@ukr.de (M.W.); 2 Department of Trauma and Hand surgery, Hospital Osnabrück, D-49076 Osnabrück, Germany; antonio.ernstberger@klinikum-os.de

**Keywords:** elderly polytrauma, anticoagulation, mortality

## Abstract

Introduction: Severely injured elderly patients have a poorer prognosis and higher mortality rates after severe trauma compared with younger patients. The aim of this study was to correlate the influence of pre-existing oral anticoagulation (OAC) and antiplatelet drugs (PAI) on the outcome of severely injured elderly patients. Methods: Using a prospective cohort study model over an 11-year period, severely injured elderly patients (≥65 years and ISS ≥ 16) were divided into two groups (no anticoagulation/platelet inhibitors: nAP and OAC/PAI). A comparison of the groups was conducted regarding injury frequency, trauma mechanism, severity of head injuries, and medication-related mortality. Results: In total, 254 out of 301 patients were analyzed (nAP: *n* = 145; OAC/PAI: *n* = 109, unknown data: *n* = 47). The most relevant injury was falling from low heights (<3 m), which led to a significantly higher number of severe injuries in patients with OAC/PAI. Patients with pre-existing OAC/PAI showed a significantly higher overall mortality rate compared to the group without (38.5% vs. 24.8%; *p* = 0.019). The severity of head injuries in OAC/PAI was also higher on average (AIS 3.7 ± 1.6 vs. 2.8 ± 1.9; *p* = 0.000). Conclusion: Pre-existing oral anticoagulation and/or platelet aggregation inhibitors are related to a higher mortality rate in elderly polytrauma patients. Low-energy trauma can lead to even more severe head injuries due to pre-existing medication than is already the case in elderly patients without OAC/PAI.

## 1. Introduction

The proportion of people 65 years and older is expected to grow in Europe to at least 30% by 2050 [1]. This change in the age structure presents new challenges for clinical routine especially in the trauma surgery sector. As the number of older patients who are admitted to emergency rooms with serious injuries is continuously rising [2], the care of seriously injured elderly patients is becoming clinical routine. A recent study [3] analyzing polytraumatized elderly patients showed that head injuries, regardless of their severity, had the biggest impact on the mortality rate in this collective. This is in accordance with the findings of Whitehouse et al., who evaluated the impact of head injuries on the outcome of severely injured older patients [4].

A relevant reason for this might be the combination between the widespread use of oral anticoagulation (OAC) [5,6] and the high risk of falling in these patients, which is known to cause serious injuries even in low-energy trauma [2]. The influence of oral anticoagulation in the pre-trauma scenario has already been investigated in several studies for head mono trauma [7,8,9,10] but with partly contradicting findings.

Nonetheless, the major concern in this vulnerable patient population is that minor bleeding could lead to a surgical emergency [11].

The purpose of this study is to analyze the influence of oral anticoagulation on the outcome of severely injured elderly patients.

## 2. Methods

For this prospective cohort study, all elderly patients admitted to our emergency department (level one trauma center) between 2006 and 2017 were recruited.

Inclusion criteria for the evaluation was an ISS ≥ 16 (Injury Severity Score) and age ≥ 65 years. The data collection was done by a study assistant (24 h/7 d) independently from the treatment algorithm. All patients with an ISS < 16 and an age < 64 years were excluded, as well as patients taking heparin.

The included patients were evaluated with regard to existing oral anticoagulation (OAC) and/or platelet aggregation inhibitors (PAI) and divided into two groups.

Group 1: Oral anticoagulation (OAC) such as non-vitamin K antagonist oral anticoagulants (NOAC) and warfarin and platelet aggregation inhibitors (PAI) such as acetylsalicylic acid (ASA) and clopidogrel.

Group 2: No oral anticoagulation or platelet aggregation inhibitors (nAP).

The demographic data are shown in Table 1.

Age, gender, ISS, severity of head injuries (specified with AIS—Abbreviated injury score for head injuries) as well as the trauma mechanisms were recorded. For both groups, a detailed breakdown of injury severity was provided.

Furthermore, the 30-day mortality of the cohort was analyzed, and in particular, the influence of OAC and PAI medication on head injuries was investigated. These results were presented in the form of Kaplan–Meier curves.

### Statistical Analysis

Kaplan–Meier curves were used to detect differences in the survival rates of both groups. A univariate data analysis was performed to compare the two groups. The Chi-Square-Test (x^2^ Test) was used to analyze binary or nominal target variables. Logistic regression analysis with the target variable “30-day mortality” followed.* p*-values of each factor were calculated as well as the corresponding 95% confidence intervals (CI). The statistical analysis (level of significance, *p* < 0.05) was carried out using SPSS software (SPSS Inc., Chicago, IL, USA).

## 3. Results

### 3.1. Demographic Data

In total, 301 patients met the inclusion criteria and 254 patients were analyzed (nAP: 145/48.2%; OAC/PAI: 109/36.2%). All patients received a full body multislice CT scan. A total of 47 patients had to be excluded because of unknown data, as the medication data were not documented due to rapid death in the emergency room (unknown data: 47/15.6%). In both groups, the majority was male (66.1%). The average age in Group 1 was a bit higher compared to Group 2 (78.3 ± 6.9 years vs. 74.9 ± 7.1 years, *p* = 0.000). The average ISS was almost identical in both groups (29.0 ± 15.4 vs. 29.1 ± 14.2; *p* = 0.971).

The number and severity of head injuries was significantly different in both groups. Patients in Group 1 showed significantly more severe head injuries (AIS ≥ 3) compared to Group 2 (81.7% vs. 63.4%). The severity of head injuries in Group 1 was also higher on average (AIS 3.7 ± 1.6 vs. 2.8 ± 1.9; *p* = 0.000).

### 3.2. Drug Classes of OAC/PAI

The majority of patients took ASA (65 patients), followed by Warfarin (38 patients). Ten patients used NOACs, and seven took clopidogrel. A total of 61 patients took PAI exclusively, and 43 patients took OAC exclusively. Five patients had a combined medication with OAC and PAI.

### 3.3. Trauma Mechanism

**Figure 1:**
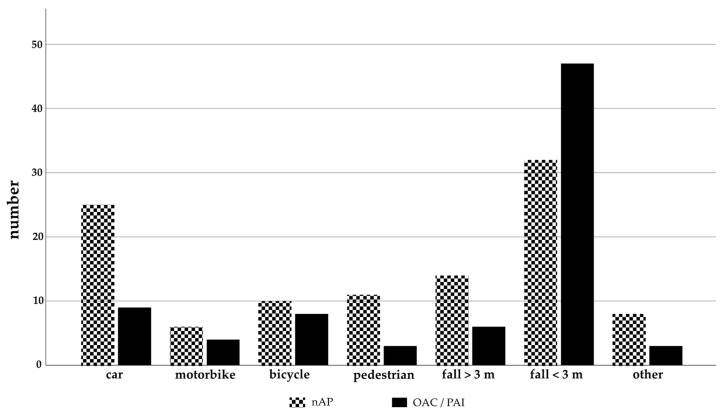
Trauma mechanism in patients with and without oral anticoagulation (OAC)/platelet aggregation inhibitors (PAI).

The main causes of severe trauma in Group 1 were falls from moderate heights (<3 m), followed by car and bicycle accidents and in decreasing order: falls from great heights (≥3 m), motorbike accidents, accidents as pedestrians, and others (Figure 1).

In Group 2, the main cause of severe trauma was also falls from moderate heights (<3 m), followed by car accidents and in decreasing order: falls from great heights (≥3 m), accidents as pedestrians, bicycle accidents, others, and motorbike accidents (Figure 1).

### 3.4. Effect of OAC/PAI On Head Injuries

**Figure 2:**
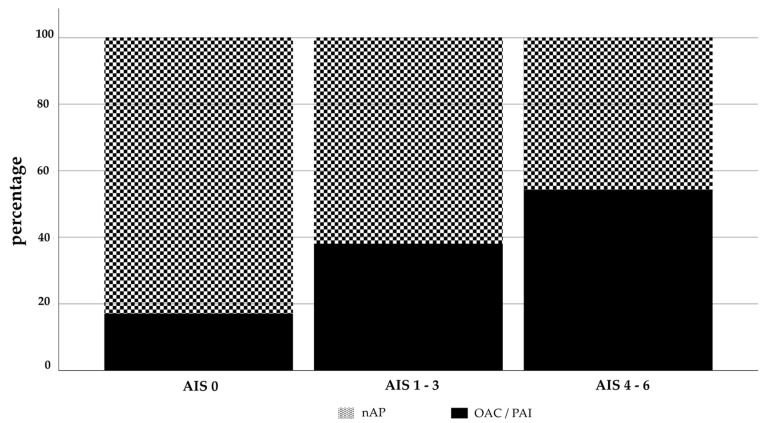
Breakdown in percent of head injuries with and without OAC/PAI depending on AIS head.

The incidence of a severe head injury increased with the use of OAC/PAI (Figure 2). The majority (>80%) of patients without head injuries (AIS 0) was found in Group 2, whereas more than 50% of patients with AIS 4–6 were in Group 1.

**Figure 3:**
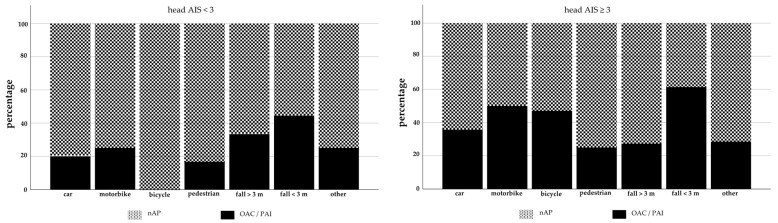
Percentage of head injuries (AIS < 3 vs. AIS ≥ 3) related to trauma mechanism.

The data (Figure 3) showed that in both groups, falls from less than three meters were the leading trauma mechanism, whereby the head injuries sustained by Group 1 were more severe (AIS 3–6).

### 3.5. 30-Day Mortality Rate

**Figure 4:**
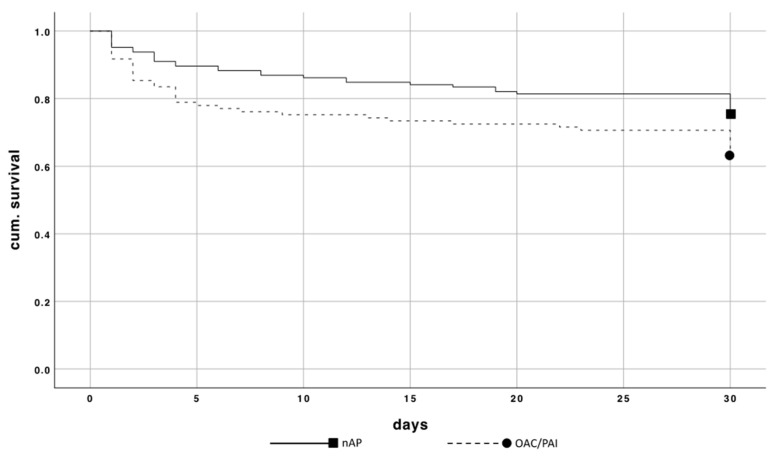
Kaplan–Meier curve of 30-day mortality comparing patients with and without OAC/PAI.

The patients of Group 1 showed a significant higher 30-day mortality rate of 38.5% compared to Group 2 with 24.8% (*p* = 0.019) (Figure 4). The relative risk of dying while taking OAC/PAI compared to patients without this medication resulted in a factor of 1.9 *(p = 0.020).*

**Figure 5:**
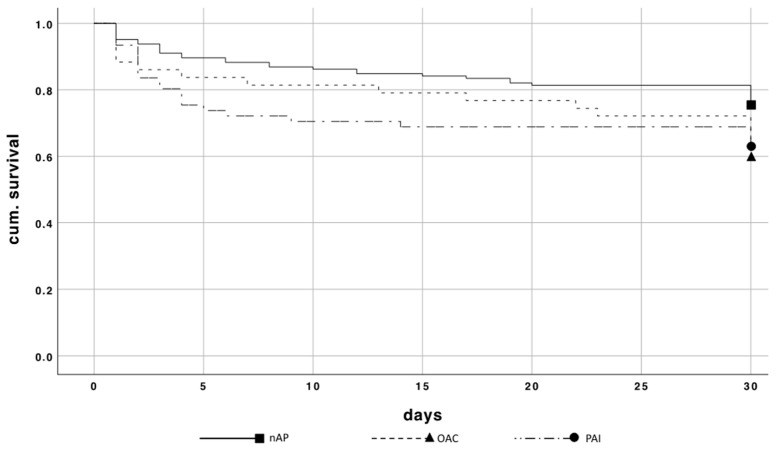
Kaplan–Meier curve of 30-day mortality comparing patients without OAC/PAI, with PAI, and with OAC.

Comparing the 30-day mortality rate of patients with PAI and OAC (Figure 5), there was a significantly higher mortality rate in the OAC group compared to PAI (41.9% vs. 37.7%; *p* = 0.04).

## 4. Discussion

In this prospective cohort study, the mechanisms and injury patterns of severe trauma in elderly people were investigated. The key findings of this study were that severely injured elderly patients taking OAC/PAI have a significantly higher 30-day mortality rate than patients without this medication and that especially low-energy traumata can lead to severe head injuries (AIS ≥ 3) in this patient population. To the best of our knowledge, this is the first study to investigate the effect of OAC/PAI in severely injured elderly patients (ISS ≥ 16).

Treatment of severely injured patients in trauma surgery is an increasing challenge in everyday life. In the last decade, new developed algorithms have noticeably improved the outcome [3,12,13,14]. However, the number of older patients who are admitted to emergency rooms with severe injuries is continuously rising [15,16,17].

As the number of elderly severely injured patients increases, so does the number of patients with pre-existing OAC/PAI medications [18].

Elderly patients per se show increased mortality rates after traumatic brain injury [19,20]. This also applies for polytraumatized elderly patients, as a recent study has shown [3]. However, a differentiation within the elderly patients with an ISS > 16 regarding pre-existing medication with OAC/PAI had not yet been investigated.

The current study showed that pre-existing medication with OAC/PAI significantly increased 30-day mortality in severely injured elderly patients. In contrast, Batey et al. described no increases in mortality after head injuries in elderly patients with OAC [18]. This is in accordance with Hwang et al. as well as Moyer et al. [21], who also showed no significant in-house mortality difference comparing patients with and without OAC/PAI before trauma. However, we have to point out that both studies investigated patients who suffered mainly from mono-traumata and not exclusively from polytrauma with an ISS ≥ 16, as in our collective. This underlines the importance of this special patient collective.

Savioli et al. describe that head trauma in itself has a negative influence on the occurrence of coagulopathies and thus on the outcome of patients [22]. This also applies to an increasing ISS. In our study group, there was no difference in ISS. In the data available to us, no conclusive distinction can be made on this topic at the level of coagulation diagnostics.

Our results correspond to the findings of Beedham et al., who described that frail, older patients often sustain head injury when they fall and are predisposed to hemorrhagic complications because of anticoagulant use and the effects of ageing [23]. As falling itself is associated with frailty [24] and especially seniors in a poorer health state are most likely to fall and therefore may have a higher risk of intracranial bleedings [25], the use of OAC/PAI in these patients is dangerous, although it may be necessary.

The comparison between OAC and PAI for in-hospital mortality in our cohort showed a higher mortality for the patients with *OAC*. This corresponds to the findings of Zhang et al., who showed OAC as a risk factor for patients with a chronic subdural hematoma in contrast to PAI [26] and Inohara et al., who found a higher mortality for patients with intracranial bleeding with prior use of OAC [27]. Nevertheless, a comparison to PAI was not investigated by both studies. Shin et al. also only compared vitamin K antagonists and NOACS. Here, a trend toward a better outcome for patients with NOACs was shown [28]. However, the results did not show a significant difference.

A recent study showed that the main cause for a severe injury (ISS ≥ 16) in elderly patients is falling from a low height (less than 3 m) [3]. This is in accordance with the findings of Lowe et al. [29]. The current study showed that this is particularly true for patients taking OAC/PAI. This is reflected in a 1.9-fold increase in the probability of death in elderly patients taking OAC/PAI medication (*p* = 0.020), which could be determined in our calculations.

In our opinion, the indication for comprehensive, ideally multislice imaging should be correspondingly generous. This is especially true for elderly patients, although Scantling et al. described no change in mortality with delayed multislice imaging in neurologically adequately assessable patients [11]. Nevertheless, based on our results of increased mortality, we recommend prompt computed tomography to rule out injuries as soon as possible in patients with pre-existing medication with OAC/PAI and suspected polytrauma injury.

There are some limitations to the present study. First, comorbidities were not taken into account due to lack of information. In order to define further recommendations for action for this sensitive patient group, this should find influence in follow-up studies. Second, the medication data of 47 patients was not documented due to rapid death in the emergency room. So, the hypothetical influence of OAC/PAI on the severity of their injuries could not be detected. It was also not possible to evaluate whether the prescribed OAC/PAI medication was correctly taken pre-trauma.

## 5. Conclusions

Pre-existing oral anticoagulation and/or platelet aggregation inhibitors are related to a higher mortality rate in elderly polytrauma patients. Low-energy trauma can lead to even more severe head injuries due to pre-existing OAC/PAI than is already the case in elderly patients without this medication.

From a trauma surgery perspective, the indication and administration of OAC and PAI should be considered very carefully and individually.

## Figures and Tables

**Table 1:** Demographic data of analyzed patients.

	OAC/PAI	nAP	*p*-Value
Number (*n*)	109	145	
Male % (*n*)	66.1 (72)	61.4 (89)	0.444
Age (years ± SD)	78.3 ± 6.9	74.9 ± 7.1	0.000 *
ISS (Ø ± SD)	29.0 ± 15.4	29.1 ± 14.2	0.971
Head AIS ≥ 3% (*n*)	81.7 (89)	63.4 (92)	0.003 *
Average AIS Head	3.7 ± 1.6	2.8 ± 1.9	0.000 *

* significant. Ø means average.

## Data Availability

Data sharing not applicable. Access to the source dataset is only permitted to employees of Department of Trauma Surgery, University Medical Centre Regensburg.
